# hMENA isoforms impact NSCLC patient outcome through fibronectin/β1 integrin axis

**DOI:** 10.1038/s41388-018-0364-3

**Published:** 2018-06-15

**Authors:** Francesca Di Modugno, Sheila Spada, Belinda Palermo, Paolo Visca, Pierluigi Iapicca, Anna Di Carlo, Barbara Antoniani, Isabella Sperduti, Anna Di Benedetto, Irene Terrenato, Marcella Mottolese, Francesco Gandolfi, Francesco Facciolo, Emily I. Chen, Martin A. Schwartz, Angela Santoni, Mina J. Bissell, Paola Nisticò

**Affiliations:** 1 Tumor Immunology and Immunotherapy Unit, IRCCS-Regina Elena National Cancer Institute, Rome, Italy; 2 Department of Molecular Medicine, University Sapienza, Rome, Italy; 3 Laboratory of Pathology, IRCCS-Regina Elena National Cancer Institute, Rome, Italy; 4 Biostatistics-Scientific Direction, IRCCS-Regina Elena National Cancer Institute, Rome, Italy; 5 Department of Physics, University Sapienza, Rome, Italy; 6 Thoracic Surgery Unit, IRCCS-Regina Elena National Cancer Institute, Rome, Italy; 7 Department of Pharmacology, Herbert Irving Comprehensive Cancer Center, Columbia University Medical Center, New York, NY USA; 8 Yale Cardiovascular Research Center, Departments of Internal Medicine, Cell Biology and Biomedical Engineering, Yale School of Medicine, New Haven, CT 06511 USA; 9 Life Sciences Division, Lawrence Berkeley National Laboratory, Berkeley, California 94720 USA

## Abstract

We demonstrated previously that the splicing of the actin regulator, hMENA, generates two alternatively expressed isoforms, hMENA^11a^ and hMENAΔv6, which have opposite functions in cell invasiveness. Their mechanisms of action have remained unclear. Here we report two major findings: (i) hMENA regulates β1 integrin expression. This was shown by depleting total hMENA, which led to loss of nuclear expression of serum response factor (SRF)-coactivator myocardin-related transcription factor 1 (MRTF-A), leading to an increase in the G-actin/F-actin ratio crucial for MRTF-A localization. This in turn inhibited SRF activity and the expression of its target gene β1 integrin. (ii) hMENA^11a^ reduces and hMENAΔv6 increases β1 integrin activation and signaling. Moreover, exogenous expression of hMENA^11a^ in hMENAΔv6-positive cancer cells dramatically reduces secretion of extracellular matrix (ECM) components, including β1 integrin ligands and metalloproteinases. On the other hand, overexpression of the pro-invasive hMENAΔv6 increases fibronectin production. In primary tumors high hMENA^11a^ correlates with low stromal fibronectin and a favorable clinical outcome of early node-negative non-small-cell lung cancer patients. These data provide new insights into the roles of hMENA^11a^ and hMENAΔv6 in the druggable β1 integrin-ECM signaling axis and allow stratification of patient risk, guiding their clinical management.

## Introduction

In order to invade, cancer cells rely on a dynamic remodeling of actin cytoskeleton [1–3].

hMENA (ENAH or MENA) along with VASP and EVL comprise the Ena/VASP family of actin regulatory proteins, which modulate cell–cell adhesion and cell migration [4]. Ena/VASP proteins share specific domains that include the EVH2 domain [5], which binds to G- and F-actin and is responsible for homo-hetero-tetramerization of Ena/VASP proteins [6]. hMENA contains a unique LERER domain that binds the α5 integrin cytoplasmic tail, affecting α5β1 signaling [7].

We initially discovered hMENA by serological analysis of recombinant cDNA expression library (SEREX) of a breast tumor with the autologous patient serum [8]. hMENA is overexpressed in primary tumors of different histological origins [9–11] compared to the normal tissues. The gene undergoes a splicing process generating multiple tissue-specific isoforms [12]. We have identified two alternatively expressed isoforms, epithelial specific hMENA^11a^ [13], and mesenchymal specific hMENAΔv6 [14]. hMENA^11a^ antagonizes whereas hMENAΔv6 promotes the invasive ability of cancer cells [10, 11, 14]. In pancreatic cancer cells, expression of hMENAΔv6, along with a lack of hMENA^11a^, is crucial for SMAD2-mediated-TGFβ signaling and invasiveness [11]. In ovarian cancer, we have recently described an essential function of hMENA/hMENAΔv6 for endothelin1/β-arrestin1-induced invadopodial activity and cancer progression [15].

We reported previously that the hMENA isoform expression pattern is a powerful prognostic factor in a couple of cancers, with high overall hMENA (including hMENAΔv6) and low hMENA^11a^ expression, identifying early non-small-cell lung cancer (NSCLC) and pancreatic cancer patients with poor prognosis [10, 11].

Changes in β1 integrin expression have been reported in mammary tumor tissues and have been associated with tissue disorganization, increased tumor aggressiveness, and metastasis [16–19]. One of the factors involved in regulation of β1 integrin expression is the serum-response transcription factor (SRF)/myocardin-related transcription factor (MRTF) complex, which binds directly to the promoter of the β1 integrin gene [20–22]. MRTF-A is retained in the cytoplasm by interacting with cytoplasmic G-actin; dissociation of this complex due to actin polymerization enables MRTF-A to translocate to the nucleus and to activate SRF-mediated gene transcription [23]. Ena/VASP proteins are well-established actin polymerases and anticapping factors that drive F-actin assembly [24, 25] and play an essential role in F-actin homeostasis [26]. Furthermore, Ena/VASP proteins and Mena specifically have previously been shown to regulate SRF activity in fibroblasts [27]. The β1 integrin signaling, through the focal adhesion kinase (FAK)-associated pathway, is one of the central mediators of cell migration and invasion [28, 29], and the activation depends on integrin conformational changes modulating the affinity for the ligands [30]. After binding of fibronectin (FN1) to α5β1, the FN1 self-association induces signaling that promotes actin cytoskeleton remodeling and cell contractility [31, 32]. In patients with breast cancer [17, 33, 34] and NSCLC [35], expression of FN1 and α5β1 was shown to be associated with poor prognosis, and in breast cancer expression of both MENA and MENA^INV^ was significantly correlated with FN, and to a lesser degree with α5 in patients with worst prognosis [7].

Here we demonstrate that hMENA controls β1 integrin expression, and provide new insights into the role of the actin regulator hMENA in the activity of the transcription factor SRF. Our findings indicate that the opposite functions of hMENA^11a^ and hMENAΔv6 in cancer cell invasion are due to their different abilities to activate β1 integrin signaling and to affect the secretion of several key extracellular matrix (ECM) proteins, including β1 integrin ligands. We propose that hMENA and its alternatively expressed isoforms are checkpoints of the targetable β1 integrin-ECM signaling pathway.

That early node-negative NSCLC patients show a prolonged disease-free survival (DFS) when expressing high hMENA^11a^/low stromal FN, offers new insights into the clinical management of these patients.

## Results

### In lung cancer hMENA correlates with β1 integrin expression and regulates nuclear MRTF-A level, SRF activity, and β1 integrin expression

We demonstrated previously that hMENA is overexpressed during lung, breast, and pancreatic ductal adenocarcinoma (PDAC) tumor progression [9–11]. Others have shown that Mena interacts with α5 integrin influencing α5β1 signaling [7]. Considering the role of β1 integrin in cancer cell invasiveness and tumor progression [33–39], we evaluated whether hMENA and its isoforms influence this integrin’s expression and signaling.

**Fig. 1:**
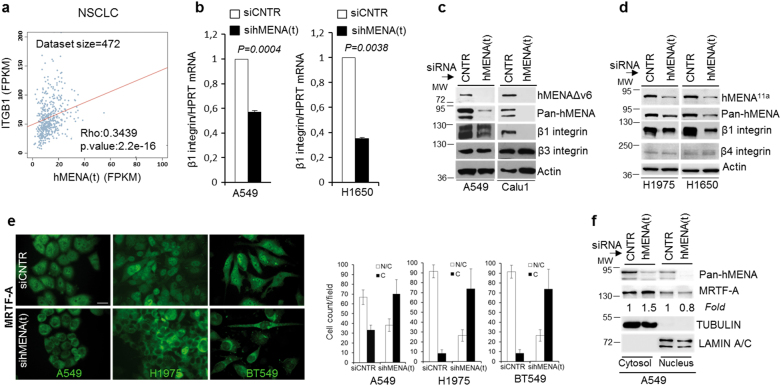
Expression of hMENA(t) correlates with β1 integrin in human NSCLC tissues and its silencing reduces β1 expression and the translocation of the SRF cofactor, MRTF-A, from the nucleus to cytoplasm in lung and breast cancer cell lines expressing hMENA^11a^ or hMENAΔv6. **a** Spearman correlation between gene expression levels of hMENA(t) (*X*-axis) and ITGB1 (*Y*-axis) in NSCLC cancer samples from TCGA. Gene expression estimates are reported as FPKM values according to the TCGA protocol. The size (number of samples/patients analyzed) of the dataset is reported on the top; statistical results are shown in the bottom-right corner. **b** qRT-PCR analysis of β1 integrin mRNA expression in the indicated cell lines transfected with control (CNTR), and hMENA(t) smart pool siRNAs. Data are reported as the mean ± SD of three independent experiments. *p* value was calculated by 2-tailed Student’s *t* test. **c**, **d** Western blot analysis of CNTR and sihMENA(t) lung tumor cell lines expressing hMENAΔv6 **(c)** or hMENA^11a^ **(d)** with the indicated antibodies. Note that β3 or β4 integrin expression is not affected by hMENA silencing. **e** Immunofluorescence staining of MRTF-A in A549, H1975, and BT549 cells transfected with nontargeting siRNA (CNTR) or total hMENA(t) siRNA. Scale bar = 30 µm, magnification ×63. Quantification of the MRTF-A subcellular distribution patterns, cytoplasmic (C) or nuclear and cytoplasmic (N/C), is shown as the mean ± SD of three independent experiments. **f** Western blot analysis of MRTF-A expression in the nucleus/cytosolic extracts of A549 cells. Anti-MRTF-A Ab immunoreactivity was determined by densitometric quantification using ImageJ, and normalized in relation to TUBULIN in the cytosolic and LAMIN A/C in the nuclear compartments

We analyzed whether the *total* hMENA, hereafter hMENA(t), transcription correlates with β1 integrin in a patient dataset from the TCGA database (https://cancergenome.nih.gov/), encompassing tumor samples from 472 NSCLC without lymph node involvement. We found a significant positive association between hMENA(t) and β1 integrin gene expression (Fig. 1a).

We explored the effect of hMENA(t) silencing on β1 integrin expression in a panel of lung cancer cell lines as well as breast cancer cells. In the invasive hMENAΔv6-positive/hMENA^11a^-negative A549, CALU1, and BT549, as well as in the hMENA^11a^-positive/hMENAΔv6-negative H1975 and H1650 cells, the hMENA(t) silencing (performed by siGENOME SMARTpool Human ENAH) led to a dramatic reduction of β1 integrin RNA and protein expression regardless of the pattern of hMENA isoforms expressed (Fig. 1b–d and Supplementary Figure 1a–c). Similar results were obtained by transiently transfecting H1650, BT549, and A549 cells with MISSION® shRNA Plasmid DNA-ENAH human (Supplementary Figure 1d). These results were confirmed by data of RNA-seq analysis performed in H1650 sihMENA(t) versus control cells, showing a significant reduction of ITGB1 (not shown). Reduction of β1 integrin occurred in parallel with a reduction of α5 integrin, as shown by Western blot (WB) analysis (Supplementary Figure 1c), and of α3 and α6, as measured by FACS analysis (Supplementary Figure 1e). However, neither β3 nor β4 integrins were inhibited, indicating specificity for β1 (Fig. 1c, d). Interestingly, we found that silencing of β1 integrin in the invasive BT549 cells did not affect hMENA(t) expression (Supplementary Figure 1b, c).

β1 integrin gene expression is regulated by SRF [20, 21], and the SRF transcriptional activity occurs after binding to the MRTF-A cofactor. We confirmed these data in BT549 cells (Supplementary Figure 2c), and then explored whether hMENA participates in the regulation of the SRF/MRTF-A complex formation. We saw that nuclear MRTF-A expression was decreased strongly by hMENA(t) depletion in all the cell lines analyzed (A549, H1975, and BT549) (Fig. 1e). Accordingly, biochemical analysis showed that hMENA(t) silencing induced a translocation of MRTF-A from the nucleus to cytoplasm (Fig. 1e, f and Supplementary Figure 2a). MRTF-A is retained in the cytoplasm by its interaction with G-actin, and the dissociation of this complex due to actin polymerization enables MRTF-A to translocate to the nucleus and activate SRF-mediated gene transcription [23]. We hypothesized that hMENA(t) affects MRTF-A subcellular localization by regulating actin dynamics. Indeed, we observed that when BT549 cells were labeled with Pan-hMENA Ab and phalloidin, hMENA(t) was localized at the tips of actin filaments. Depletion of hMENA(t) resulted in abrogation of stress fiber, actin reorganization, and a dramatic cell shape change (Supplementary Figure 2b). The assessment of G-actin/F-actin ratio, reflecting the balance between actin polymerization and depolymerization, showed a decrease in F-actin with a significant increase in the G-actin/F-actin ratio in hMENA(t)-silenced BT549 cells with respect to control cells (Supplementary Figure 2d).

The expression of SRF was not influenced by hMENA(t) silencing in BT549 cells (Supplementary Figure 2e). However, the analysis of the effect of hMENA(t) depletion on SRF activity by serum response element (SRE)-reporter assay experiments showed that BT549 cells had a significant reduction of SRF activity, comparable to the effect observed using the SRF inhibitor CCG1423 in relation to control cells (Supplementary Figure 2f).

These data show that hMENA affects MRTF-A nuclear translocation and SRF activity independently of the pattern of expressed isoforms, suggesting that hMENA sustains β1 integrin expression by affecting SRF activity.

### hMENA^11a^ and hMENAΔv6 isoforms regulate β1 integrin activation in opposite directions

Previously we reported that the alternatively expressed hMENA^11a^ and hMENAΔv6 isoforms have opposite functions in cell invasiveness of breast, lung, and pancreatic tumors [10, 11, 14]. This general mechanism is of great clinical relevance in early NSCLC patients, where high Pan-hMENA and low hMENA^11a^ were the only independent predictors of shorter DFS [10]. We suspected that the opposite roles of these isoforms on cancer cell invasiveness may be dictated by a different effect on β1 integrin activation. Since we have previously reported the pro-invasive role of hMENAΔv6 in lung cancer cells [10], we silenced SRF and found the abrogation of this hMENAΔv6 function in A549 cells (Supplementary Figure 3).

We examined the role of hMENA isoforms in β1 integrin activation in invasive hMENA/hMENAΔv6-positive cancer cell lines. A clear colocalization of hMENA/hMENAΔv6 and the active form of β1 integrin was revealed by immunofluorescence analysis using Pan-hMENA Ab and 9EG7 Ab, specific for the extended (active) conformation of β1 integrin [40]. The hMENA(t) fluorescence intensity is enriched in the areas of active β1 integrin clusters, which were completely abrogated by hMENA(t) silencing as evidenced by 9EG7 staining (Supplementary Figure 4a, b). To assess whether this dramatic effect is related to the reduction of β1 integrin expression caused by hMENA(t) silencing, we evaluated the ratio between the active β1 integrin (9EG7) and the total β1 integrin expression (TS2-16). TS2-16 binds to the head of β1 integrin either in bent or in extended conformation [30]. We found that hMENA(t) depletion strongly reduced the percentage of active β1 integrin with respect to the total level of β1 integrin, in both breast BT549 and lung A549 cancer cells (Supplementary Figure 4c, d). Importantly, the silencing of VASP, the other Ena/VASP member, did not reduce the expression and activation of β1 integrin (Supplementary Figure 4e, f).

**Fig. 2:**
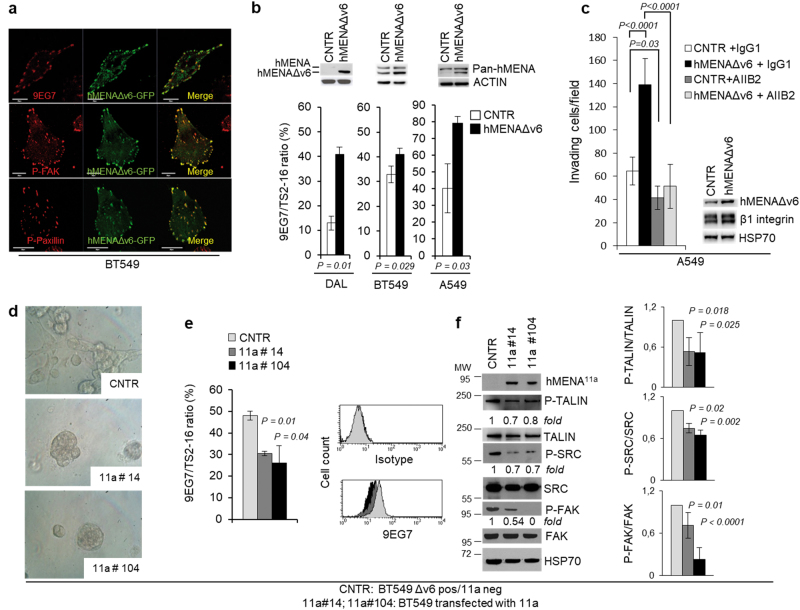
hMENAΔv6 localizes at focal adhesions and its overexpression increases the β1 integrin activation, whereas hMENA^11a^ transfection in hMENAΔv6-expressing BT549 cells induces morphological changes, inhibits β1 integrin activation, and the downstream signaling pathway. **a** Confocal analysis with the indicated antibodies (red) of BT549 cells transfected with hMENAΔv6-GFP (green). Magnification ×63. Scale bar = 20 µm. **b** Percentage of active cell surface β1 integrin (9EG7) with respect to total β1 integrin (TS2-16) analyzed by flow cytometry in DAL, BT549, and A549 cells transfected with empty vector (CNTR) or hMENAΔv6 showing that hMENAΔv6 transfection increases the activation of β1 integrin. Data are reported as the mean ± SEM of three independent experiments. *p* value was calculated by 2-tailed Student’s *t* test. The upper panel shows Western blot analysis with Pan-hMENA Ab. **c** Matrigel invasion assay performed on A549 cells (50,000 cells; 24 h of invasion), transiently transfected with the empty vector (CNTR) or hMENAΔv6, and treated with β1 integrin blocking antibody AIIB2 or with IgG1 isotype control toward serum. The assay was repeated three times, performed in triplicate each time. Standard deviations are indicated. *p* value was calculated by 2-tailed Student’s *t* test. Inset: WB analysis of the A549 cells to verify hMENAΔv6 transfection. **d** Phase-contrast microscopy images of BT549 cells transfected with empty vector (CNTR) and hMENA^11a^-expressing cell clones (#14 and #104) when grown for 10 days in 3D on lrECM. Magnification ×40. **e** Percentage of active β1 integrin (9EG7) with respect to total β1 integrin (TS2-16) analyzed by flow cytometry in BT549 CNTR (hMENA^11a^ negative/hMENAΔv6 positive) or hMENA^11a^-expressing clones, showing that hMENA^11a^ transfection reduces the activation of β1 integrin. Data are reported as the mean ± SD of three independent experiments. *p* value was calculated by 2-tailed Student’s *t* test. Representative histograms of flow cytometric staining for 9EG7 in BT549 CNTR or hMENA^11a^--expressing clones are shown. **f** WB analysis of BT549 CNTR or hMENA^11a^-expressing clones with the indicated antibodies, showing that hMENA^11a^ transfection reduces the phosphorylation of β1 integrin signaling partners, TALIN, SRC, and FAK. Immunoreactivity was determined by ImageJ and phosphoproteins were normalized in comparison with the respective total proteins (TALIN, SRC, and FAK). Numbers indicate the fold changes. The fold reduction of phoshorylated protein expression in hMENA^11a^-expressing clones is reported on the right. Data are reported as the mean ± SD of three independent experiments

To confirm the specific role of hMENAΔv6 isoform in β1 integrin activation, we transiently transfected this isoform in BT549 cells. The hMENAΔv6 isoform tagged with GFP localized at focal adhesion with the active β1 integrin and with P-FAK and P-paxillin (Fig. 2a). Furthermore, we observed a significant increase in β1 integrin activation (Fig. 2b). This effect is even more evident when we stably transfected DAL, a breast cancer cell line that, unlike BT549, is poorly invasive and expresses undetectable levels of hMENA isoforms [14] and low levels of β1 integrin (Fig. 2b). We observed the hMENAΔv6-mediated increase in β1 integrin activation also in the A549 lung cancer cell line (Fig. 2b). The confocal analysis show a higher intensity of P-FAK and P-paxillin in the cells transfected with hMENAΔv6-GFP with respect to the neighboring untransfected cells (Supplementary Figure 5a, b).

To assess whether β1 integrin activation contributes to hMENAΔv6 pro-invasive function, we treated the A549 cells that were transiently transfected with hMENAΔv6, with the β1 integrin blocking antibody AIIB2. Matrigel invasion assay demonstrated that β1 integrin inhibition hampered cell invasion and impeded the pro-invasive effect of hMENAΔv6 transfection (Fig. 2c), suggesting that hMENAΔv6 exerts its pro-invasive role by activating β1 integrin. We evaluated whether the dominant anti-invasive hMENA^11a^ [14] exerted an inhibitory effect on β1 integrin activation. hMENA^11a^-transfected BT549 cells were cloned and, when grown in 3D on laminin-rich ECM (lrECM), showed a “mass” morphology compared to the “stellate” morphology of BT549 control cells (Fig. 2d for clones #14 and #104). In addition, we observed a significant reduction of the extended β1 integrin conformation staining using 9EG7 in the two hMENA^11a^ clones analyzed (Fig. 2e) with respect to control. Interestingly, we found a significant reduction of the phosphorylation of talin and of the β1 integrin downstream signaling partners, FAK, SRC (Fig. 2f), and paxillin (Supplementary Figure 6a, b), suggesting that hMENA^11a^ inhibits the β1 integrin activation pathway. Notably, this occurred also in BT549 cells transduced with the splicing regulator ESRP1, which includes the 11a exon [41] (Supplementary Figure 6a). On the contrary, the transfection of hMENAΔv6 in DAL and A549 cells increases the phosphorylation status of talin and FAK (Supplementary Figure 5c, d).

### Proteomic analysis reveals that hMENA^11a^ expression modifies the ECM composition

Our observation that the anti-invasive hMENA^11a^ isoform reduced β1 integrin activity prompted us to perform proteomic analysis of the secretome of two BT549 hMENA^11a^-transfected clones and control cells (CNTR).

**Fig. 3:**
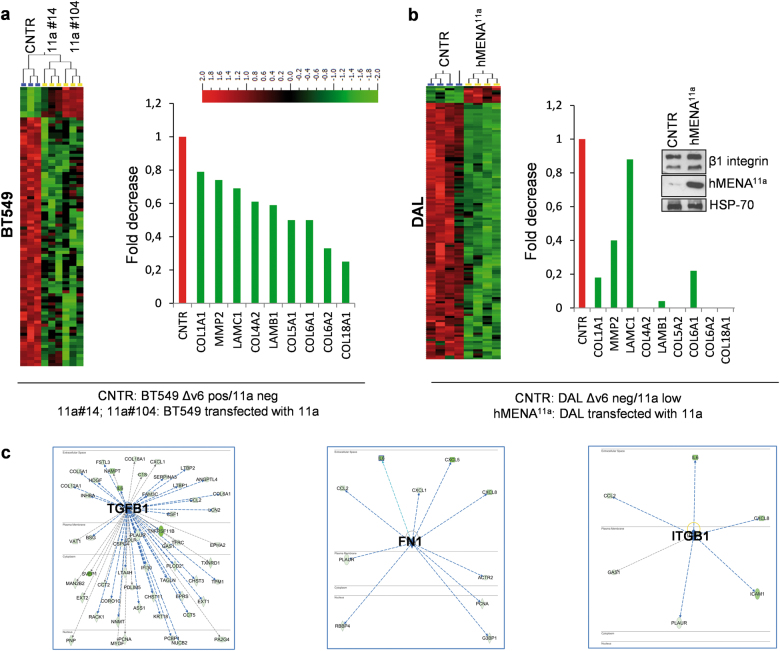
hMENA^11a^ transfection significantly reduces ECM components in the secretome of breast tumor cells. **a** Heat map of proteins found to be differentially secreted in BT549 CNTR cells expressing hMENAΔv6 and in hMENA^11a^-expressing cell clones (#14 and #104) (left). Analysis was conducted on three biological replicates. *T*-tests and Benjamini and Hochberg’s FDR Control Algorithm was applied to the resulting large-scale data and FDR threshold used to generate the differentially expressed protein list. Fold change rank of proteins associated with ECM composition (right). **b** Heat map of proteins found to be differentially secreted across DAL CNTR cells and hMENA^11a^ stable transfectants (left). Analysis was conducted on four biological replicates. Fold change rank of proteins associated with ECM composition also identified in BT549 cells (right). Western blot analysis of DAL CNTR and DAL/hMENA^11a^ cells with the indicated Abs (inset). **c** Three networks identified by upstream regulator algorithm with predicted *z*-score action using the Ingenuity Pathway Analysis (IPA)

Among the 1721 quantifiable proteins identified in the conditioned medium (CM) of the cells, we observed 373 proteins (10% false discovery rate (FDR), *q* = 0.1) and 255 proteins (5% FDR, *q* = 0.05), which were significantly different in the two hMENA^11a^-transfected clones compared to control cells (Fig. 3a). Upstream regulator analysis from Ingenuity Pathway (Qiagen, MD) of differentially expressed proteins identifies the down-regulation of several important signaling pathways in the BT549/hMENA^11a^ clones. TGFβ1 signaling pathways were the most significant (activation *z*-score = −4389, *p* value = 112E-20), along with the pathways of FN1 (activation *z*-score = −2472, *p* value = 179E-06) and β1 integrin (activation *z*-score = −2182, *p* value = 0.000282) (Fig. 3c).

Many of these proteins are identified as ECM molecules (by GeneOntology) and are associated with β1 integrin activation (Fig. 3a). Moreover, among the significantly reduced secreted proteins we found the colony-stimulating factor 1 (CSF1), in agreement with previous data in MTLn3 mammary tumors reporting that increased MENA^11a^ expression correlates with decreased expression of CSF1 [42].

We confirmed these data in DAL cancer cell line, the only cell line that expresses undetectable hMENA(t), stably transfected with hMENA^11a^. Secretome analysis showed that among 1811 quantifiable proteins (Supplementary Table 1), 1324 were significantly different between the transfected hMENA^11a^ and the control CM (Fig. 3b). Among these differentially expressed proteins, we showed the significant reduction of β1 integrin ligands in both BT549 hMENA^11a^ and DAL hMENA^11a^ compared to the CM from their respective control cells. Apart from ECM components, we found also that hMENA^11a^-transfected cells showed a reduced secretion of ECM remodeling enzymes, such as matrix metalloproteinase 2 (MMP2) (Fig. 3a, b), in agreement with our recent observation in pancreatic cancer cell lines [11]. To gain insight into the mechanism underlying the effects of the hMENA^11a^ on β1 integrin ligand secretion and to analyze whether hMENAΔv6 plays an opposite role, we analyzed the expression of FN1 in DAL cells transfected with hMENA^11a^ or hMENAΔv6. The FN1 transcript is strongly induced by hMENAΔv6, but not affected by hMENA^11a^ (Supplementary Figure 7d). At the protein level, results showed that transfection of hMENA^11a^ reduces whereas hMENAΔv6 increases the level of FN1 protein both in the CM as well as in the DAL cell lysates (Supplementary Figure 7a–c). At the functional level, the reduction of β1 integrin activation observed in the hMENA^11a^ /BT549 cell clones is abrogated when the assay is performed on cells grown for 1 h on FN1 (Supplementary Figure 7e). These data suggest that hMENA^11a^ has a dominant anti-invasive role due to its ability to inhibit both the secretion of pro-invasive β1 integrin ligands such as fibronectin and ECM degrading enzymes such as MMP2.

### High hMENA^11a^ along with low stromal FN1 expression prolongs the disease-free survival of early node-negative NSCLC patients

**Fig. 4:**
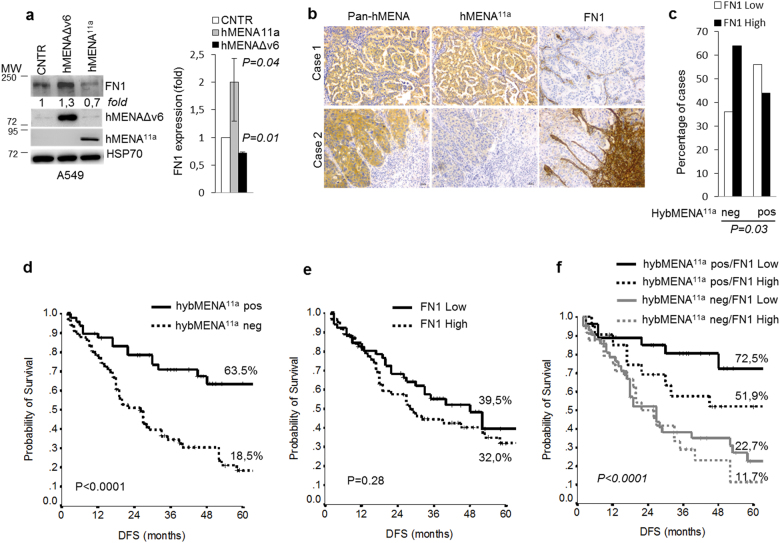
hMENAΔv6 increases and hMENA^11a^ reduces the FN1 expression in NSCLC cell lines and low level of FN1 in the stroma of high hMENA^11a^ expressing tumors is a good prognostic factor in early node-negative NSCLC patients. **a** Western blot analysis of A549 cells transfected with empty vector (CNTR), hMENAΔv6, and hMENA^11a^ with the indicated Abs. The fold increase or reduction of FN1 protein expression in hMENAΔv6- or hMENA^11a^-transfected cells of different experiments is reported on the right. Data are reported as the mean ± SD of three independent experiments. **b** Consecutive sections of representative cases of NSCLC decorated with Pan-hMENA, hMENA^11a^, and FN1 Abs. All magnification values are × 20. Scale bar = 30 µm. **c** Immunohistochemical characterization of 114 node-negative NSCLC tissues showing that hybMENA^11a^ pos (high hMENA^11a^ /low hMENA [t]) cases more frequently express low FN1 level in the stroma. *p* value was estimated with Fisher Exact test. **d**–**f** Kaplan–Meier estimate of disease-free survival (DFS) of resected, node-negative, NSCLC patients according to dichotomized hybMENA^11a^ and stromal FN1 expression

**Fig. 5:**
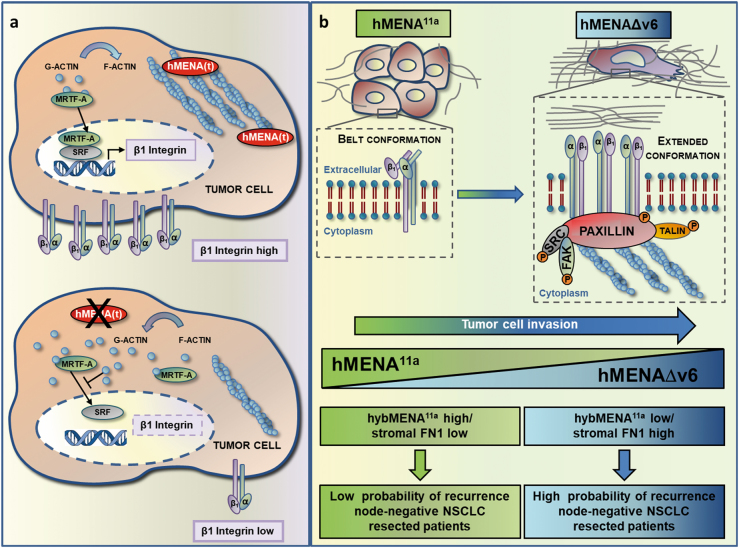
Working model: **a** hMENA(t) regulates nuclear MRTF-A localization, SRF activity, and β1 integrin expression. Tumor cells overexpressing hMENA(t) show nuclear MRTF-A localization, active SRF, and overexpress β1 integrin. hMENA(t) depletion (below) inactivates SRF and reduces β1 integrin expression by increasing the G/F-actin ratio and retaining MRTF-A in the cytoplasm. **b** hMENA^11a^ and hMENAΔv6 isoforms have opposite functions in the β1 integrin activity, ECM composition and is associated with the clinical outcome of node-negative NSCLC patients

Previously we noted that hMENA^11a^ is a useful prognostic factor in node-negative NSCLC patients [10]. Given the results of mass spectrometry analysis showing that a high ratio of hMENA^11a^/hMENAΔv6 expression decreases the secretion of a protein cluster belonging to the FN1 pathway, and given the relevance of FN1 expression in lung cancer [43], we evaluated the effect of hMENA^11a^ and hMENAΔv6 transfection on FN1 expression in lung cancer cell lines. Results showed that transfection of hMENA^11a^ reduces whereas hMENAΔv6 increases the level of FN1 protein (Fig. 4a). To confirm the clinical relevance of these experimental results, we analyzed FN1 expression in 114 tissues of early node-negative NSCLC patients (patient characteristics are reported in Supplementary Table 2), in parallel with Pan-hMENA and hMENA^11a^ staining, scored as hybMENA^11a^-positive (which denotes cases with hMENA^11a^ high/hMENA(t) low) or -negative (all other cases including tumors expressing hMENAΔv6) [10]. We found that FN1 expression in the tumor stroma is distributed differently in hybMENA^11a^-positive or -negative tumors (Fig. 4b, c) (*p* = 0.03). High FN1 was significantly more expressed in the hybMENA^11a^-negative group, which included tumors expressing hMENAΔv6. In contrast, hybMENA^11a^-positive tumors express essentially a low FN1 level in the stroma, in agreement with the proteomic data obtained for cancer cell lines. Of clinical relevance, Kaplan–Meier curves for DFS, according to the combination of the two variables, indicate that 72.5% of patients with hybMENA^11a^-positive and low FN1 are disease free at 5 years (*p* < 0.0001), whereas when we considered only hybMENA^11a^ expression, we found that 63.5% of the patients were disease free at 5 years (Fig. 4d–f). These data confirm, but also strengthen the conclusion that the combination of high hMENA^11a^ and low stromal FN1 is a promising prognostic factor in early node-negative NSCLC patients (Fig. 5).

## Discussion

The bidirectional transmission of information among actin cytoskeleton, integrins, and ECM has been widely studied in cancer invasion [1], and an increase in the β1 integrin level has clearly been shown to increase the growth rate of breast cancer cells [37] and be directly associated with malignant phenotype in three-dimensional assays [16, 44, 33, 38, 45–47]. Knowledge of the link between cytoskeletal actin dynamics and gene expression is growing continuously, and our present findings indicate that the actin cytoskeleton regulatory protein, hMENA, controls the level of expression of β1, and that the alternatively expressed hMENA ^11a^ and hMENAΔv6 isoforms inhibit or activate β1 integrin signaling, respectively.

Analyzing a cohort of TGCA node-negative NSCLC patient data, we demonstrated a significant correlation between hMENA and β1 integrin expression (Fig. 1a). To attack the mechanistic basis linking hMENA and β1 integrin expression, we performed a series of molecular and biochemical studies in a panel of NSCLC and breast cancer cell lines. We demonstrated that hMENA controls β1 integrin expression, but does not affect the expression of other integrins such as β3 and β4 integrins (Fig. 1c, d) implicated in cancer cell motility and tumor progression.

β1 integrin is a target gene of SRF [20], crucial in the communication between the actin cytoskeleton and the genome, representing a model of the link between cytoarchitecture and gene activity [48, 49]. Our data that the depletion of all hMENA isoforms inhibits the nuclear expression of the cofactor MRTF-A (Fig. 1e) indicate a role of hMENA in SRF/MRTF-A circuit. This is in line with our previous data reporting that hMENA and its isoforms may affect the nuclear localization of transcription factors such as FOXO3A [50] and SMAD2 [11]. Indeed, the silencing of hMENA isoforms in PDAC invasive cancer cell lines abrogated SMAD2 phosphorylation and its nuclear localization, impairing the TGFβ-mediated EMT [11], suggesting that hMENA may control the activity of this and other transcription factors. Importantly, we showed that hMENA silencing increases the G-actin/F-actin ratio, crucial for MRTF-A subcellular localization, and reduces the activity of SRF (Supplementary Figure 2d, f). Although further studies are needed to unequivocally prove some of the above, we can hypothesize that, by affecting the G-actin/F-actin ratio, hMENA may be of importance in the control of nuclear transcriptional activity that favors expression of genes involved in tumor cell motility and tumor progression. The Rho-GTPase Cdc42 was shown to similarly promote cancer cell transendothelial migration by regulating β1 integrin gene transcription through SRF-mediated activation [22].

Integrin activation is coordinated by actin cytoskeleton dynamics [51]. We have demonstrated previously that the alternatively spliced hMENA isoforms impact cytoarchitecture [10] and are linked to malignant progression in a 3D model of human breast cancer progression [14]. Using a panel of NSCLC and breast cell lines, we have also found that hMENA regulates β1 integrin, independently of the pattern of hMENA isoform expression (Fig. 1b–d). To search for the mechanisms by which hMENA ^11a^ exerts an anti-invasive and hMENAΔv6- a pro-invasive function in the absence of hMENA^11a^, we focused on the role of these two isoforms in β1 integrin activation.

The β1 activation status depends on integrin conformational changes that modulate the affinity for the ligands, the recruitment of adaptor proteins, and the phosphorylation of downstream signaling partners (i.e., FAK, SRC, and paxillin) [52]. Using antibodies able to specifically bind the different conformations of β1 integrin [30], we showed that the different roles of hMENA isoforms are dictated by the status of β1 integrin activation. In fact, our gain and loss of function experiments (Fig. 2 and Supplementary Figures 4 and 5) show that hMENAΔv6 induces active β1 integrin expression. The observation that hMENAΔv6 localizes to clusters of active β1 integrin, FAK, and paxillin at focal adhesions (Fig. 2a) sustains its functional participation. In cell invasion assays, we demonstrated that β1 integrin inhibition hampered cell invasion and impeded the pro-invasive effect of hMENAΔv6 transfection (Fig. 2c), indicating that the pro-invasive function of hMENAΔv6 is mediated by its ability to activate β1 integrin. Conversely, we showed that transfection of the anti-invasive hMENA^11a^ dramatically reduced activation of β1 integrin and phosphorylation of talin, reported to be required for β1 integrin activation [53], FAK, SRC (Fig. 2f), and paxillin. We obtained similar results when expression of hMENA^11a^ was induced *via* its splicing regulator ESRP1 (Supplementary Figure 6a).

We hypothesize that hMENA^11a^ and hMENAΔv6, respectively, inhibit or induce the release of the bent inactive conformation of β1 integrin by interacting with different adaptor molecules. This has been reported for VASP, the other Ena/VASP family member, that when associates with Rap1-GTP-interacting adaptor molecule (RIAM) at focal adhesions increases the talin binding to β1 integrin and its inside-out activation [54]. Whereas in the current studies VASP silencing does not affect β1 integrin activation (Supplementary Figure 4e, f), the hypothesis that the two isoforms may differentially influence the inside-out β1 integrin activation is supported by the increase/decrease of talin phosphorylation induced by transfection of either hMENAΔv6 or hMENA^11a^, respectively (Supplementary Figure 5c, d and Fig. 2f). We could also speculate that the two isoforms may bind with different affinities to α5 integrin, causing a diverse α5β1 receptor maturation as shown for MENA^INV^ [7], a MENA isoform described as relevant in cancer cell invasiveness, but not restricted to mesenchymal-like invasive cancer cells as we have demonstrated for hMENAΔv6. However, further studies are needed to clarify whether hMENAΔv6 has an increased or distinct activity compared to the coexpressed canonical hMENA.

The findings show clearly that hMENA^11a^ transfection in cells that do not contain this isoform inhibits different ECM proteins. In view of the recent published results on the novel role for Mena in the regulation of local translation of specific mRNAs in developing axons, including FN1 mRNA, it could be posited that hMENA^11a^ restrains the posttranscriptional effect of hMENA [55]. Our data show that hMENA^11a^ inhibits the secretion of β1 integrin ligand collagens, laminin, and fibronectin (Fig. 3a, b), suggesting a mechanism underlying the hMENA^11a^-mediated reduction of β1 integrin activation, supported by the abrogation of this reduction when the assay is performed on cells grown on FN1 (Supplementary Figure 7e). Indeed, it was shown [56] that binding of ECM ligands to the high-affinity conformation of β1 integrin induces outside-in activation with the recruitment of a signaling cascade leading to actin cytoskeleton rearrangements. The clear correlation between the isoform composition and invasion, as well as changes in the pattern of ECM proteins and TGFβ pathway, strongly point to the clinical relevance of our findings. Another conclusion of this study is the need to pay serious attention to the patterns of protein expression in the stroma of node-negative NSCLC patients in both prognosis and treatment. Thus a combination of high hMENA^11a^ and low stromal FN1 in the lung cancer tissues would predict a favorable outcome and the opposite would be true for high hMENAΔv6 and high FN1. This would create new opportunities for the ongoing debate of the clinical management of these patients.

## Materials and methods

Details regarding materials and cell lines and cultures and information relative to patients and tissue specimens can be found in the Supplementary Materials and Methods.

### TCGA analysis

The analysis of correlation between hMENA and β1 integrin gene expression was carried out in patient dataset from the TCGA database (https://cancergenome.nih.gov/), encompassing 472 samples of NSCLC without lymph node involvement. The analysis is described in detail in the Supplementary Information.

### Immunohistochemistry

Pan-hMENA (mouse, clone 21, 610693, BD Biosciences), hMENA^11a^ [13], and FN1 (mouse, IST-4, F0916, Sigma-Aldrich) immunoreactions were revealed by Bond Polymer Refine Detection (Leica Biosystem, Milan, Italy) on an automated autostainer (BondTM Max, Leica).

Staining for hMENA(t) and hMENA^11a^ was evaluated as previously reported [10] on whole tissue sections.

Stromal FN1 was scored using a scale from 0 to 3 (score 0: no staining, score 1: weak, 2: moderate, and 3: strong). For statistical analysis grades 0–1 were merged and labeled as “Low,” whereas grades 2–3 were combined and labeled as “High.”

Evaluation of the immunohistochemistry results was performed independently by two investigators (MM and PV) blinded to patient data.

### Small interfering RNA (siRNA)

SMARTpool small interfering RNAs (GEHealthcare, Dharmacon, Lafayette, CO, USA) were used for hMENA(t), β1 integrin, and SRF silencing as previously reported [11] and detailed in supplementary materials and methods. hMENA knockdown was also obtained by transient transfection of MISSION® shRNA Plasmid DNA-ENAH human-TRCN0000303614 (Sigma-Aldrich) targeting the 3’UTR region.

### Transfections

A 3 × 10^5^ cells/well were plated in six-well plates and the next day transfected with 2.5 μg/ml *hMENA*^11a^, *hMENAΔv6* cDNA (untagged or GFP-tagged), or with vector alone (pcDNA3), using LipofectAMINE2000 (Invitrogen, Carlsbad, CA). Stable transfectants were obtained by selecting transfected cells with 500 μg/ml of G418 (Invitrogen). Clones of BT549 breast cancer cells retrovirally infected with pMSCV-hMENA^11a^ vector were selected from the bulk cell culture by limiting dilution. BT549 ESRP1 transduced cells were obtained as previously reported [14].

### WB analysis

Protein extracts and WB analysis were performed as previously reported [14]. Nuclear and cytoplasmic fractions were obtained using NE-PER Nuclear Cytoplasmic Extraction Reagent kit (Pierce, Rockford, IL, USA).

Antibodies used are reported in Supplementary Material and Methods.

Densitometric quantitation of antibody immunoreactivity was determined by Image J 1.49 v program (NIH) and normalized as reported in figure legends.

### RNA extraction and real-time PCR

RNA extraction and real-time PCR were performed as previously described [14] and detailed information are reported in Supplementary Materials and Methods.

### SRF activity, luciferase reporter assay

Luciferase reporter assay was performed using the Dual-Glo Luciferase Assay Kit (Promega). Cells seeded in a 96-well plate in triplicates were concurrently transfected with hMENA(t) or CNTR siRNAs, and SRE-driven firefly luciferase reporter plasmid and Renilla luciferase pRL-TK control plasmid (SRE Reporter assay kit, Quigen) with Cignal dual-luciferase SRE Reporter. After 72 h, 75 µl of Dual-Glo Luciferase Reagent was added to the culture medium and after 10 min the firefly luciferase and Renilla luminescence were measured.

Untranfected BT549 cells treated with CCG1423 (Cayman Chemical, Ann Arbor MI, USA) were used as control of SRF activity inhibition.

Data are shown as relative firefly luciferase expression normalized to Renilla luciferase.

### Immunofluorescence

Cells transfected with siRNAs or hMENA isoform vectors were analyzed by immunofluorescence as previously reported [14] and as detailed in the Supplementary Materials and Methods.

### G-actin/F-actin in vivo assay

The amount of filamentous actin (F-actin) content versus free globular-actin (G-actin) content was determined using G-actin/F-actin in vivo assay Biochem kit (Cytoskeleton, Denver, CO). The samples obtained were analyzed for actin quantification by SDS-PAGE and WB.

### Flow cytometry

Flow cytometry was performed on cells grown on uncoated plates or on FN1 (25 µg/ml) coated culture plates for 1 h and mechanically detached from the plates by the use of a scraper. Washing steps and antibody incubations were performed in 20 mM Hepes, pH 7.4, 150 mM NaCl, 3% dialyzed FCS, 0.1% azide, containing either 1 mM each Mg2+ and Ca2+ or 0.2 mM Mn2+ (as a positive control of β1 integrin activation). Cells were stained with 9EG7 mAb or TS2-16 mAb, for 30 min at room temperature. After washing, cells were incubated in FITC-labeled antirat (Bethyl Laboratories, Montgomery, TX, USA) or antimouse (Cappel Laboratories, West Chester, PA, USA) antibodies, respectively. Cells were acquired on a BD FACSCantoII flow cytometer (BD Biosciences) and analyzed using FACSDiva and CellQuest software (BD Biosciences). Dead cells were excluded by propidium iodide staining (MP Biomedicals, Santa Ana, CA, USA). The percentage of positive staining of 9EG7 mAb (corrected for background by subtracting the percentage of the antibody control isotype) was divided by the percentage of positive staining for the TS2-16 mAb (corrected for background) to measure the active/total β1 integrin ratio.

### Cell invasion assay

Cell invasion assay was performed as previously reported [10] in Matrigel invasion chamber (BD Biocoat Matrigel invasion chamber, BD Biosciences) in serum-free medium, or in serum-free medium containing 0.5 mg/ml of AIIB2 mAb (Aragen Bioscience, Morgan Hill, CA, USA) or control Isotype IgG1 (eBioscience, CA, USA).

### Analysis of the secretome by liquid chromatography coupled with tandem mass spectrometry (LC-MS/MS)

For MS analysis, cells were grown in RPMI 1640 medium and CM collected after 48 h of culture; protease and proteinase inhibitors were added immediately. CM was centrifuged at 3500 rpm for 30 min to remove cell debris and the supernatant was concentrated using the Amicon Ultra 15, Ultracel-3K centrifugation device (Thermo Fisher, San Josè, CA, USA) until the volume was reduced to ~100 ul. The protein concentration was determined using the Qubit Protein Assay (Thermo Fisher). Detailed description of the analysis is reported in Supplementary Materials and Methods.

### Statistics

Descriptive statistics were calculated for all the variables. Categorical variables were reported as frequencies and percentage values, while continuous variables were summarized through mean values and their relative standard deviation (SD) or standard error of the mean (SEM). Unpaired Student’s *t*-test was used when appropriate. The correlation analysis was performed with Spearman test.

The Pearson’s chi-Square test or Fisher Exact test, when appropriate, was applied to assess the relationship between biological parameters. DFS was calculated by the Kaplan–Meier product limit method from the date of the surgery until relapse or death. Significance was defined at the *p* < 0.05 level. All the analyses were performed with SPSS statistical software version 21.0 (SPSS Inc., Chicago IL, USA).

## Electronic supplementary material

Supplementary Materials and Methods

Supplementary Figures

Supplementary Figure Legends

Supplementary Table 1

Supplementary Table 2
