# Supplementary material for: hMENA isoforms impact NSCLC patient outcome through fibronectin/β1 integrin axis
Source: Oncogene. 2018 Jun 15;37(42):5605–17. doi: 10.1038/s41388-018-0364-3 (PMC6193944; doi:10.1038/s41388-018-0364-3)
Supplement: Supplementary file 1 — Supplementary Materials and Methods [file 41388_2018_364_MOESM1_ESM.docx]

**Supplementary Materials and Methods**

**Materials**

Dithiothreitol (DTT), acetonitrile (ACN), ammonium bicarbonate, trifluoretic acids (TFA), and iodoacetamide (IAA) were from Thermo Fisher Scientific (Waltham, MA, USA). Trypsin Gold, mass spectrometry grade, was from Promega (Madison, WI, USA). Nanopure water was prepared with use of Milli-Q water purification system (Millipore, Billerica, MA, USA).

**Cell lines and 3D cultures**

The human breast (BT549) and lung (A549, H1975, CALU-1) cancer cell lines were purchased from American Type Culture Collection (ATCC, Rockville, MD). DAL breast cancer cells were developed in our laboratory from the ascitic fluid of a breast cancer patient.^1^ Cells were cultured in RPMI 1640 medium (Gibco, Invitrogen, Pisley, UK) supplemented with 1% (vol/vol) Glutamine and 10% (vol/vol) inactivated fetal bovine serum at 37°C in 5% CO_2_ 95% air. For 3D cell cultures, cells (1 × 10^5^) were seeded on top of a growth factor reduced reconstituted basement membrane Matrigel (BD Pharmingen) containing 5% laminin-rich extracellular matrix and plated in six-well plates. Cancer cell lines were authenticated by BMR Genomics (Padova, Italy). All cell lines were routinely checked for Mycoplasma using Mycoplasma PCR Reagent set (Euroclone, Italy).

**TGCA analysis**

Gene expression quantification files had previously been generated according to the HTSeq^2^– FPKM (Fragments per Kilobase per Millions of mapped reads) computational workflow and downloaded from the Genomic Data Common (https://gdc-portal.nci.nih.gov) portal. Correlation of gene expression was assessed using a Spearman correlation index test across patients of the same tumor type.

**Patients**

NSCLC patients resected with curative intent at the Regina Elena National Cancer Institute between 2001 and 2006 and without pathological lymph-node involvement (N0) were considered eligible for the prognostic analysis (Table S1). Follow-up data were obtained from hospital charts and by corresponding with the referring physicians, analyzed, and reported according to Shuster and coauthors.^3^ The study was reviewed and approved by the ethics committee of the Regina Elena National Cancer Institute, and written informed consent was obtained from all patients prior to inclusion in the study.

**Small interfering RNA (siRNA)**

Small interfering RNA performed as 3 × 10^5^ cells/well in exponential growth phase were plated in 6-well plates. The next day, cells were transfected with 20 nmol/L of hMENA(t)-specific pooled siRNA duplexes (siGENOME SMARTpool Human ENAH), 4 nmol/L of β1 integrin-specific pooled siRNA duplexes (ON-TARGETplus SMARTpool Human ITGB1), 10 nmol/l SRF-specific pooled siRNA duplexes (ON-TARGETplus SMARTpool Human SRF) or 20nmol/l of ON-TARGETplus Nontargeting Control Pool (GEHealthcare, Dharmacon, Lafayette, CO, USA) using Lipofectamine® RNAiMAX Transfection Reagent (Invitrogen, Carlsbad, CA, USA) according to the manufacturer’s protocol. The specific effect of hMENA(t) silencing was validated using transient transfection of MISSION® shRNA Plasmid DNA - ENAH human - TRCN0000303614 (Sigma-Aldrich). The effects of silencing were evaluated at 72h from the transfection.

**Western Blot analysis. Antibodies employed**

The following primary antibodies were used: rabbit hMENAΔv6 (ref. 4), rabbit Pan-hMENA (HPA028696, Sigma-Aldrich), mouse hMENA^11a^ (ref. 4), mouse β1 (18/cd29, 610468), mouse β3 integrin (1/Integrin Beta 3 CHAIN, 611141), mouse P-FAK (Y397) [14/FAK(Y397), 611723], (BD Biosciences), mouse β4 integrin (450-11A, gently provided by Dr. Rita Falcioni, Regina Elena National Cancer Institute, Rome), rabbit SRF (G-20, sc-335), mouse α5 integrin (B-4, sc-166681), mouse HSP-70 (W27, sc-24), rabbit Histone H3 (FL-136, sc-10809), mouse Lamin A/C (346, sc-7293) (**Santa Cruz Biotechnology, Inc.** CA, USA), rabbit MRTF (HPA030782), mouse Actin (clone AC-40, A4700), mouse Talin (clone 8d4, **T3287**), mouse Fibronectin (IST-4, F0916, Sigma-Aldrich), rabbit Tubulin (11H10, 2125), rabbit FAK (3285), rabbit P-Paxillin (Tyr 118, 2541), rabbit Paxillin (2542), rabbit P-SRC (Tyr 416, 2101), rabbit SRC (2108) (Cell Signaling Technology, Danvers, MA, USA), rabbit P-Talin (Ser-425, TP4171, ECM Biosciences, Versailles, KY, USA).

**RNA extraction and real-time PCR**

Total RNA was isolated from cells using TRIzol reagent (Invitrogen). Reverse transcription was carried out using first-strand cDNA synthesis kit (GE Healthcare). Quantitative RT-PCR (qRT-PCR) reactions were performed in triplicates using KAPA PROBE FAST Universal qPCR kit (KAPA Biosistems, MA, USA) in ABI Prism 7500 Real-time PCR instrument (Applied Biosystems, Princeton, NJ, USA). TaqMan Gene Expression Assays (from Applied Biosystems) were used for amplification and quantification of ENAH, β1 integrin and FN1 genes and of human hypoxanthine phosphoribosyltransferase 1 gene (HPRT1), used as an endogenous control. The comparative Ct method (2−ΔΔ/Ct method) was used to determine changes in relative levels of different genes.

**Immunofluorescence**

Cells transfected with siRNAs or hMENA isoform vectors were plated on coverlips precoated with Gelatin 2% and grown for 1 h or 24 h before fixing and permeabilizing as previously reported^4^ and stained with: rabbit Pan-hMENA (HPA028696, Sigma-Aldrich), rabbit MRTF (HPA030782), mouse P-FAK (Tyr 397, 611807, BD Biosciences), rabbit P-Paxillin (Tyr 118, 2541, Cell Signaling Technology) Abs. For active β1 integrin staining (rat 9EG7, 550531, BD Bioscences), cells were incubated with the antibody for 30’ at 4°C before fixation. For positive control of β1 integrin activation cells were pre-incubated with a solution containing 1 mM MnCl2. After multiple washes with PBS, the cells were incubated with Alexa Fluor 594 or Alexa Fluor 488 anti-mouse, anti-rat or anti-rabbit secondary antibody (Thermo Fisher Scientific) for 30 min at room temperature. Actin filaments were stained with Alexa Fluor™ 594 Phalloidin (Thermo Fisher Scientific). The coverslips were washed and mounted with the VECTASHIELD® MOUNTING MEDIUM with DAPI (Vector Laboratories). Immunofluorescence was analyzed by Leica DM IRE2 microscopy with Leica FW 4000 software (Leica, Solms, Germany) or with Zeiss LSM 510 Meta confocal laser scanning microscope equipped with a 60X/1.23 NA oil immersion objective. As laser (488 and 514 nm), and HeNe laser (543 nm) were used to excite the fluorophores. The Zeiss Zen control software (Zeiss, Germany) was used for image analysis. For quantification of hMENA(t) in the β1 integrin clusters, images were quantified by creating a mask for areas (region of interest) of active β1 integrin based on the 9EG7 staining. hMENA(t) fluorescence intensity in masked areas was compared to that in the whole cell (n=10) to evaluate enrichment of hMENA(t) in the β1 integrin clusters. Immunofluorescence intensity of P-FAK and P-Paxillin was quantified on images where both untransfected or hMENAΔv6-GFP transfected cells were present, by creating a mask around the single cells. Immunofluorescence intensity of P-FAK and P-Paxillin of the cells GFP positive (n=10) was compared to that of the neighbor cells GFP negative (n=10). To quantify colocalizations we used the Pearson's correlation coefficient (R). Cells (n = 10) of selected samples were analyzed by using PSC Colocalization plug-in (ImageJ-NIH;^5^).

**Analysis of the Secretome by liquid chromatography coupled with tandem mass spectrometry (LC-MS/MS)**

*In Solution Protein Digestion*

5 μg of proteins were reduced, alkylated, and digested in 50 μL of 200 ng Trypsin Gold reconstituted in 50 mM ammonium bicarbonate at 37°C overnight. After the digestion was complete, the peptide mix was centrifuged subsequently for 30 min at 14,000 rpm, and the cleared supernatants were transferred to fresh tubes to be dried and resuspended in 0.1% TFA for subsequent peptide fractionation using the Pierce High pH Reversed-Phase Peptide Fractionation Kit (Thermo Fisher). Peptide fractionations were collected for LC-MS/MS analysis.

*LC-MS/MS Analysis*

The dried peptide mix was reconstituted in a solution of 2% acetonitrile (ACN), 2% formic acid (FA) for MS analysis. Peptides were loaded directly onto a 2 cm C18 PepMap pre-column by an autosampler (Thermo Scientific), which was coupled to a 50 cm EASY-Spray C18 analytical column (Thermo Scientific). Peptides were eluted from the column using a Dionex Ultimate 3000 Nano LC system with a 2 min gradient from 2% buffer B to 5% buffer B (100% acetonitrile, 0.1% formic acid), followed by a 65 min gradient from 5% buffer B to 20% buffer B and a 15 min gradient from 20% to 30% buffer B. The gradient was switched from 30% to 85% buffer B over 1 min and held constant for 3 min. Finally, the gradient was changed from 85% buffer B to 98% buffer A (100% water, 0.1% formic acid) over 1 min, and then held constant at 98% buffer A for 20 more minutes. The application of a 2.0 kV distal voltage electrosprayed the eluting peptides directly into the Thermo Fusion Tribrid mass spectrometer equipped with an EASY-Spray source (Thermo Scientific). Mass spectrometer-scanning functions and HPLC gradients were controlled by the Xcalibur data system (Thermo Finnigan, San Jose, CA). MS data were acquired in the Fourier Transforming (FT) at 120,000 resolution from m/z 400-1600. CID MS/MS were acquired in the IT on 2^+^ and higher charge state ions for 3 sec duty cycles.

*Database search and interpretation of MS/MS data*

Acquired MS data were searched against a human UniProt database (released on 4/27/2016 and common contaminants were added) using Proteome Discoverer 1.4 with fixed modifications of carbamidomethyl on Cysteine and possible oxidation on methionine. The Proteome Discoverer probability-based scoring system rates the relevance of the best matches found by the SEQUEST algorithm. The peptide mass search tolerance was set to 10ppm. A minimum sequence length of 7 amino acids residues was required. Only fully tryptic peptides were considered. To calculate confidence levels and FDR, Proteome Discoverer generates a decoy database containing reverse sequences of the non-decoy protein database and performs the search against this concatenated database (non-decoy + decoy). Scaffold (Proteome Software) was used to visualize searched results. The discriminant score was set to be less than 1% FDR, which are determined based on the number of accepted decoy database proteins to generate protein lists for this study. Spectral counts were used for semi-quantitative comparisons of protein abundance among samples.

*Statistics & bioinformatics analysis*

Qlucore Omics Explorer (Qlucore AB, Sweden) was used to perform statistical analysis of differentially expressed proteins. We applied t-tests and Benjamini and Hochberg's FDR Control Algorithm^6-8^ to the resulting large-scale data. FDR threshold used to generate the differentially expressed protein list is specified in the result section. Ingenuity Pathway Analysis (Qiagen, MD) was used to perform data mining of the proteomics data.

**References**

1. Nisticò P, De Berardinis P, Morrone S, Alonzi T, Buono C, Venturo I, et al. Generation and characterization of two human alpha/beta T cell clones. Recognizing autologous breast tumor cells through an HLA- and TCR/CD3-independent pathway. *J Clin Invest*  1994; **94**:1426-1431.

2. Anders S, Pyl PT, Huber W. HTSeq—a python framework to work with high-throughput sequencing data. *Bioinformatics* 2015; **31**: 166-169.

3. Shuster JJ. Median follow-up in clinical trials. *J Clin Oncol* 1991; **9** :191-192.

4. Di Modugno F, Iapicca P, Boudreau A, Mottolese M, Terrenato I, Perracchio L, et al. Splicing program of hMENA produces a novel isoform associated with invasive, mesenchymal-like breast tumors. *Proc Natl Acad Sci* USA 2012; **109**: 19280-19285.

5. French AP, Mills S, Swarup R, Bennett MJ, Pridmore TP. Colocalization of fluorescent markers in confocal microscope images of plant cells. *Nat Protoc* 2008; **3**: 619–28.

6. Storey JD, Taylor JE, Siegmund D. Strong control, conservative point estimation and simultaneous conservative consistency of false discovery rates: a unified approach. *J R Statist Soc B* 2004; **66**, Part 1, pp. 187–205.

7. Benjamini Y, Krieger AM, Yekutieli D. Adaptive linear step-up procedures that control the false discovery rate. *Biometrika* 2006; **93**: 491-507.

8. Gavrilov Y, Benjamini Y, Sarkar S. An adaptive step-down procedure with proven FDR control under independence. *Ann. Statist* 2009; **37:** 619–629.
