# Supplementary material for: hMENA isoforms impact NSCLC patient outcome through fibronectin/β1 integrin axis
Source: Oncogene. 2018 Jun 15;37(42):5605–17. doi: 10.1038/s41388-018-0364-3 (PMC6193944; doi:10.1038/s41388-018-0364-3)
Supplement: Supplementary file 2 — Supplementary Figures [file 41388_2018_364_MOESM2_ESM.pdf]

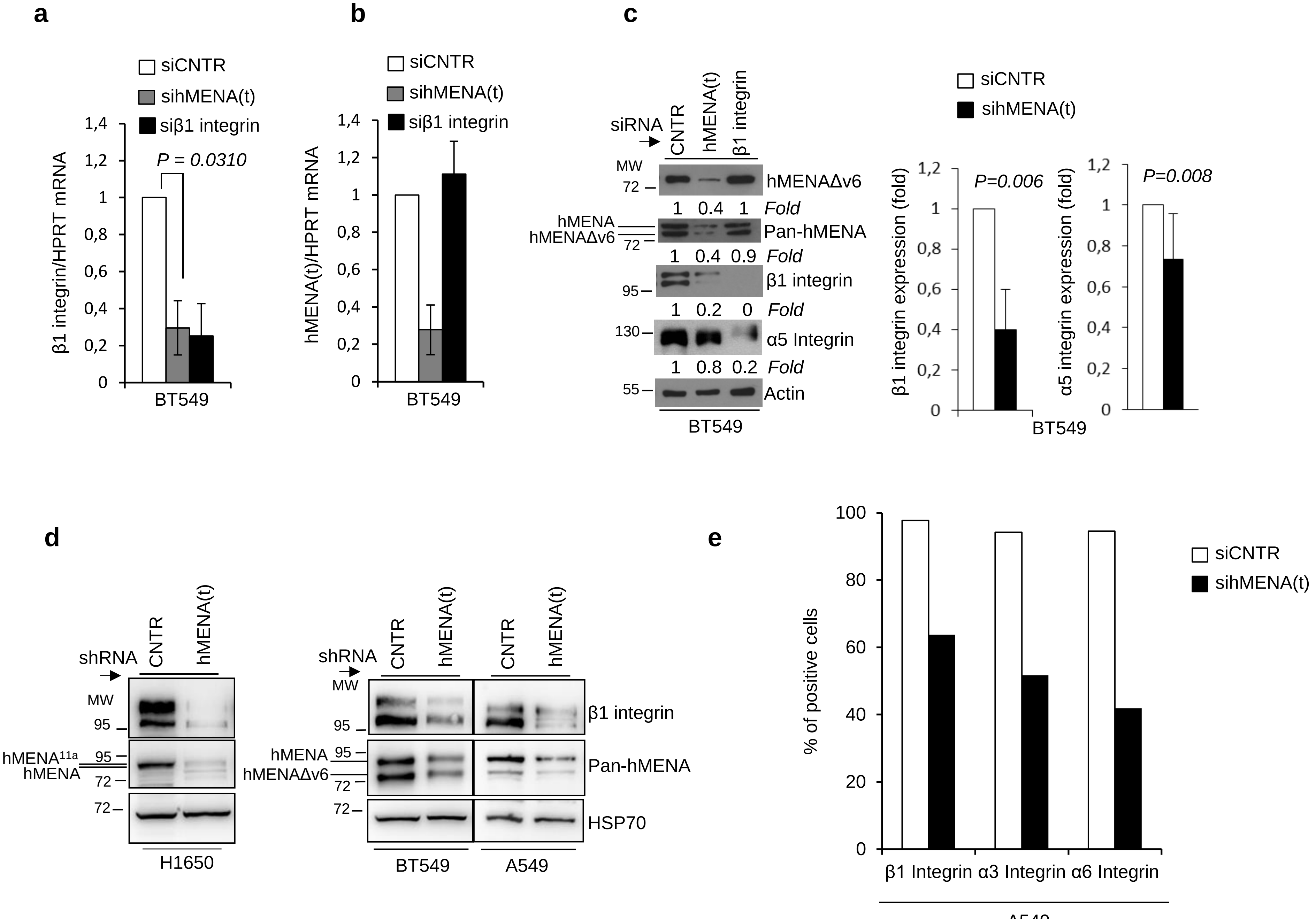

Supplementary Figure 1.

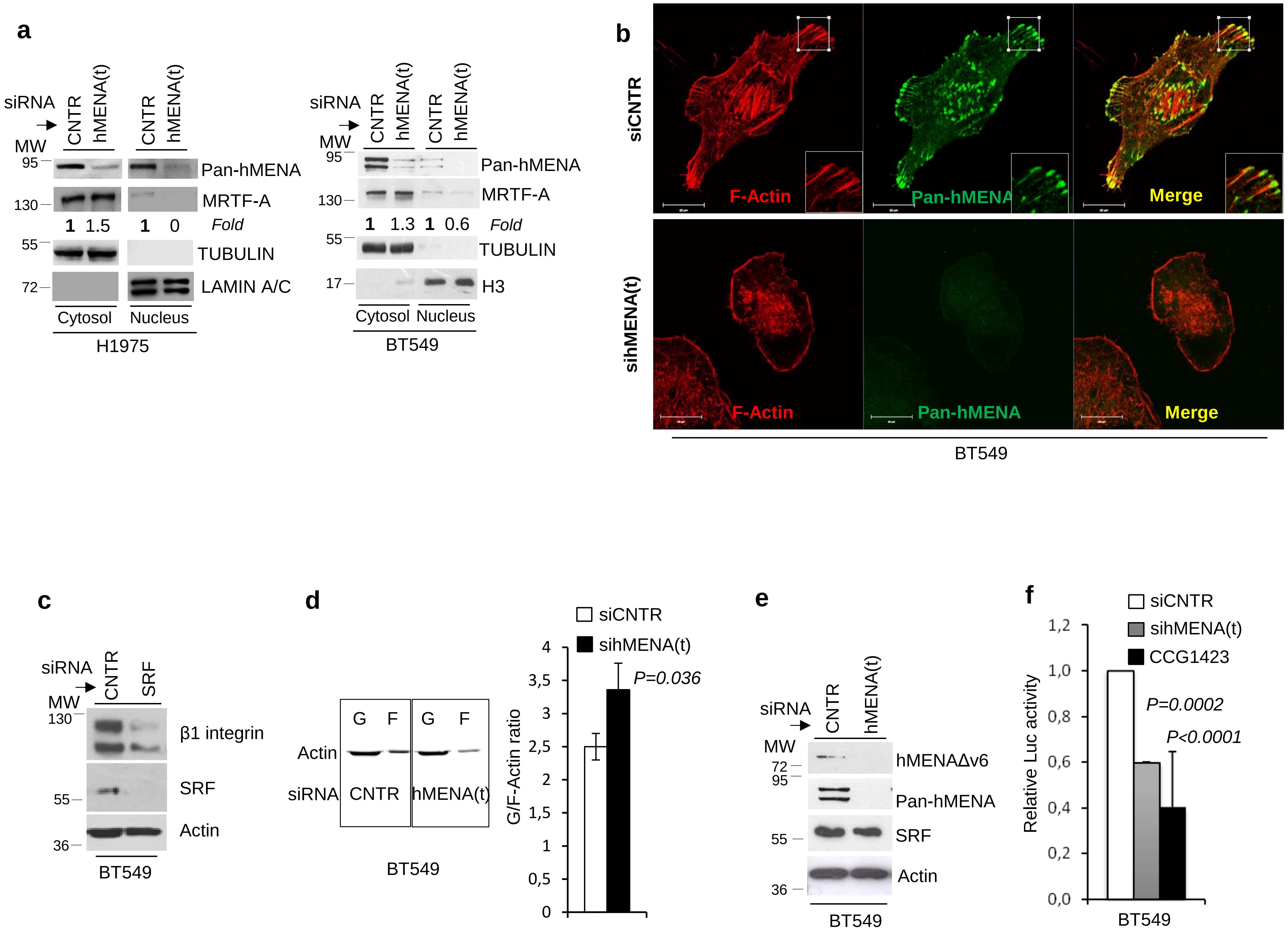

Supplementary Figure 2.

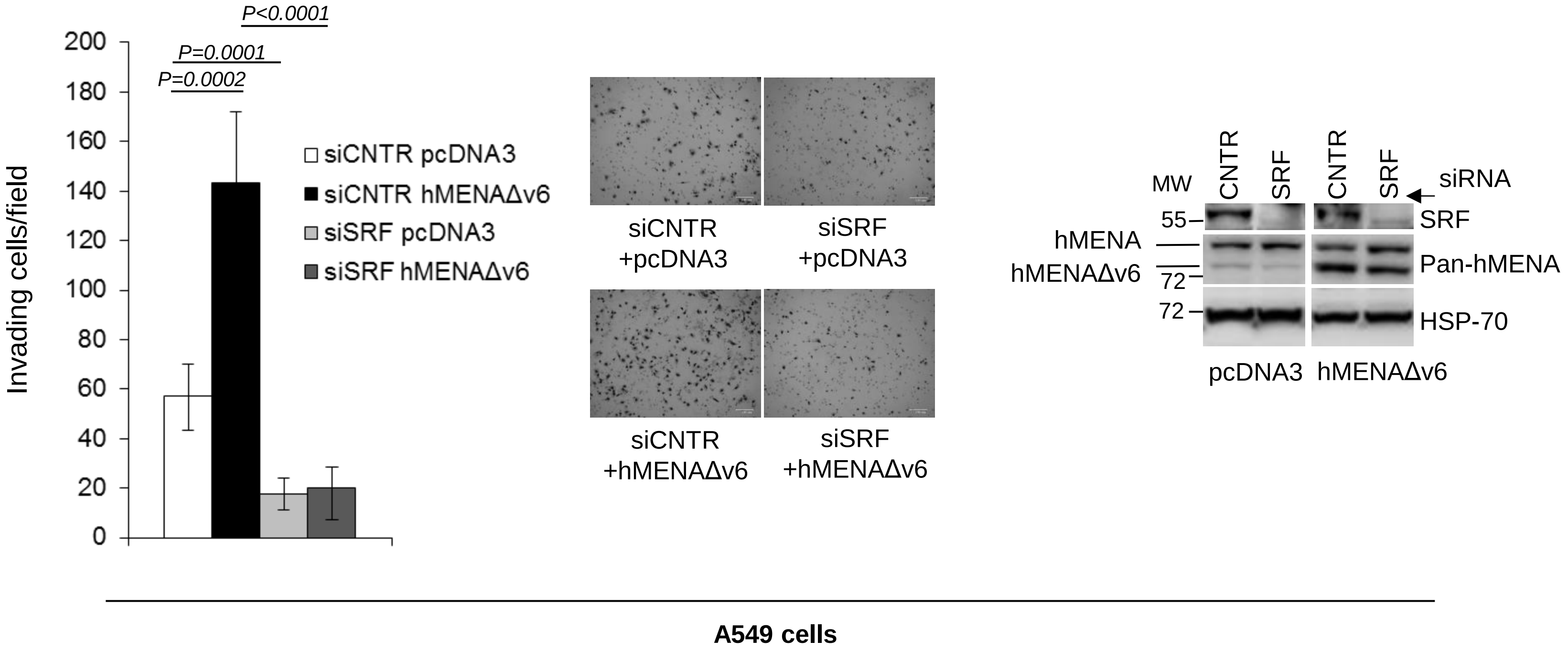

Supplementary Figure 3.

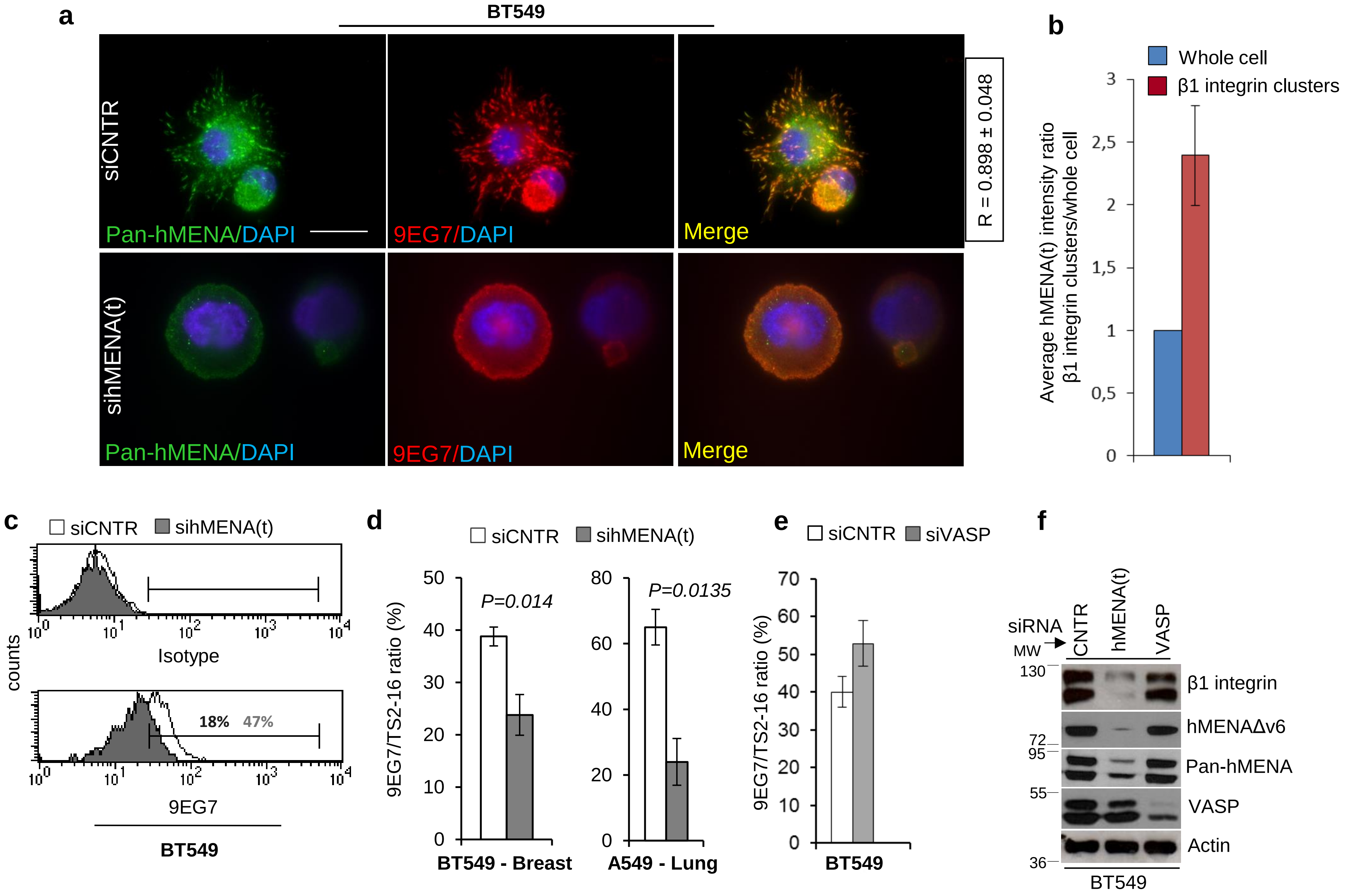

Supplementary Figure 4.

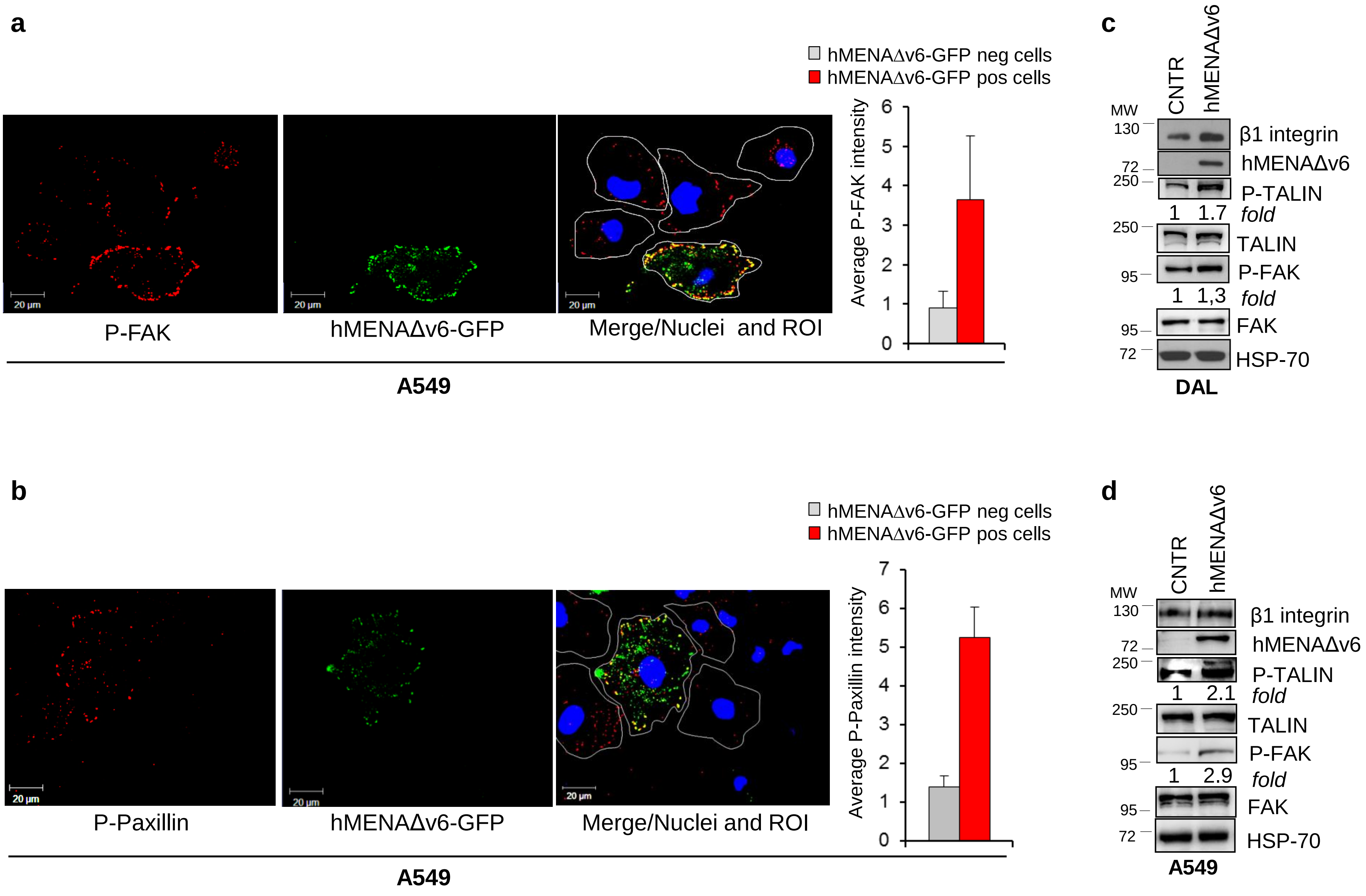

Supplementary Figure 5.

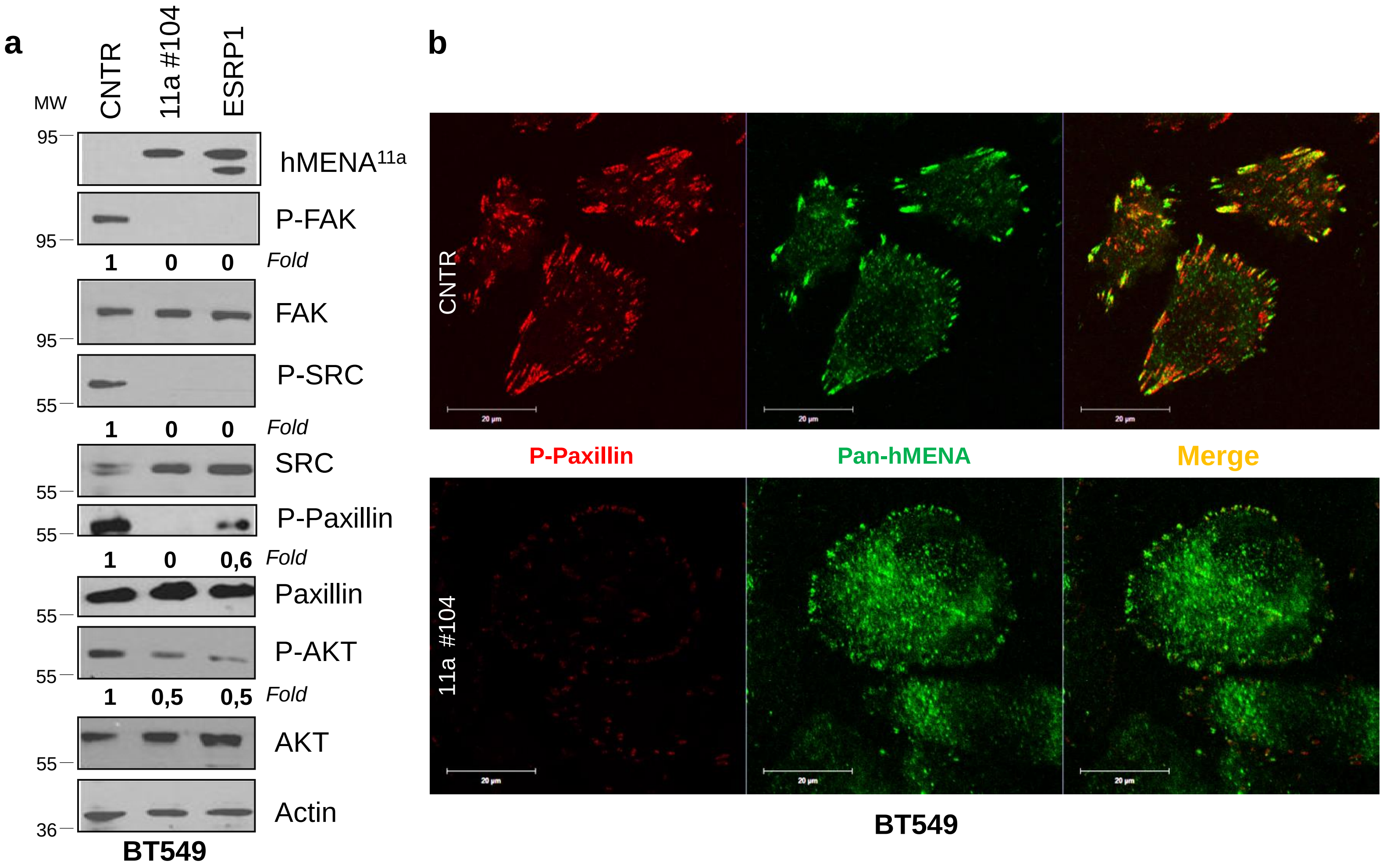

Supplementary Figure 6.

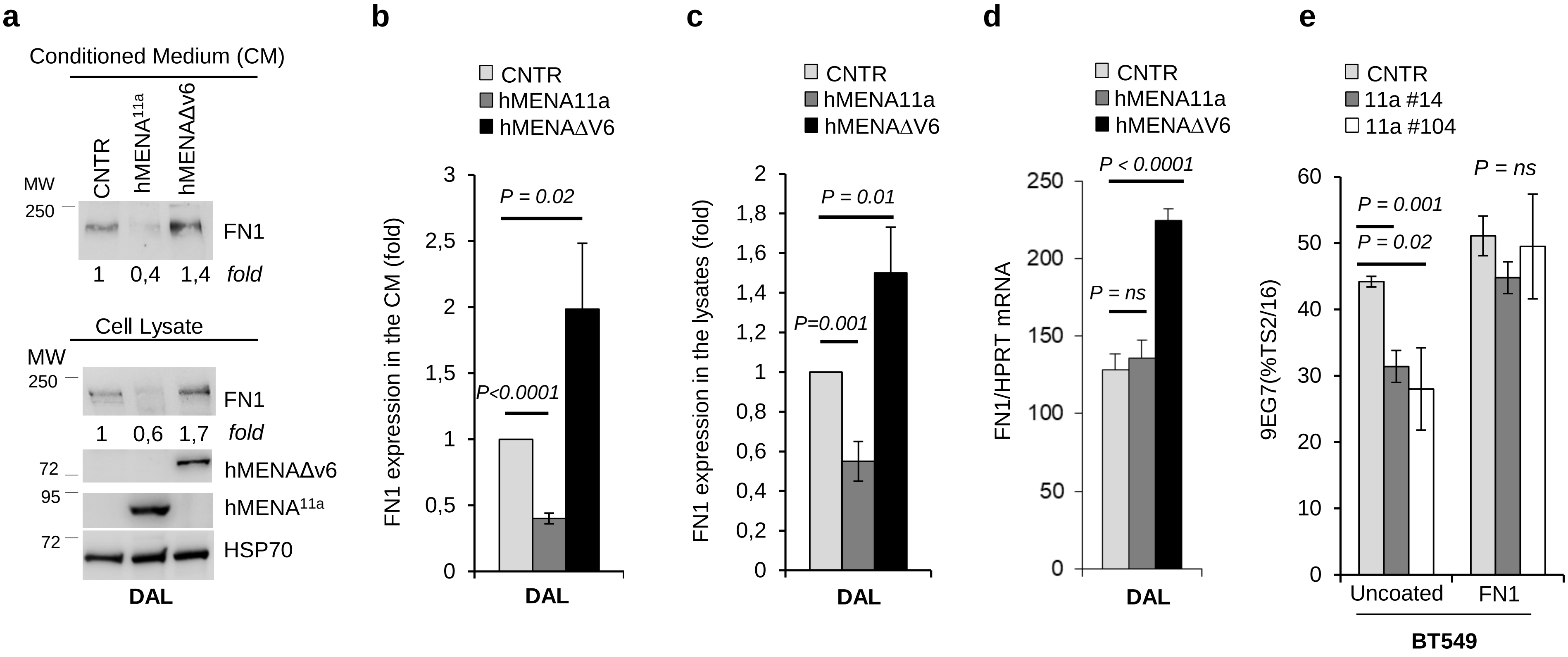

Supplementary Figure 7.
