# Supplementary material for: hMENA isoforms impact NSCLC patient outcome through fibronectin/β1 integrin axis
Source: Oncogene. 2018 Jun 15;37(42):5605–17. doi: 10.1038/s41388-018-0364-3 (PMC6193944; doi:10.1038/s41388-018-0364-3)
Supplement: Supplementary file 3 — Supplementary Figure Legends [file 41388_2018_364_MOESM3_ESM.docx]

**Supplementary Figure Legends**

**Supplementary Figure 1.** hMENA(t) silencing reduces the expression of β1 integrin and of its dimerization partners, α3 and α6 in cancer cells. **(a-b)** qRT-PCR analysis of β1 integrin (a) and hMENA (b) mRNA expression in the BT549 cells transfected with control (CNTR), hMENA(t) and β1 integrin smart pool siRNAs. Data are reported as the mean ± SD of three independent experiments. P value was calculated by 2-tailed Student’s t test. **(c)** Western blot analysis of CNTR, sihMENA(t) and siβ1 integrin BT549 cells, with the indicated Abs. Note that β1 integrin depletion does not affect the expression of hMENA(t). The fold reduction of β1 and α5 integrin protein expression in sihMENA(t) BT549 cells of different experiments is reported on the right. Anti-β1 integrin and anti-α5 integrin immunoreactivity was determined by densitometric quantification using ImageJ, and normalized in relation to the Actin. Data are reported as the mean ± SD of three independent experiments. **(d)** Western blot analysis of H1650, BT549 and A549 cells transfected with MISSION® TRC2 pLKO.5-puro Empty Vector Control Plasmid DNA (CNTR), or MISSION® shRNA Plasmid DNA - ENAH human - TRCN0000303614 (Sigma-Aldrich) targeting the 3’UTR ENAH region, with the indicated Abs. **(e)** Percentage of cells positive for β1, α3 and α6 integrin surface expression, analyzed by flow cytometry in A549 cells transfected with non-targeting (CNTR) or hMENA(t) siRNAs.

**Supplementary Figure 2.** hMENA(t) silencing induces translocation of the SRF co-factor, MRTF-A, from the nucleus to cytoplasm, reduces F-Actin content and SRF activity. **(a)** Western blot analysis of MRTF-A expression in nucleus/cytosolic extracts of the H1975 and BT549 cells. Anti-MRTF-A Ab immunoreactivity was determined by densitometric quantification using ImageJ, and normalized in relation to TUBULIN in the cytosolic LAMIN A/C or H3 and in the nuclear compartments. **(b)** Confocal analysis of BT549 cells transfected with non-targeting siRNA (CNTR) or hMENA(t) siRNA, labeled with anti-Pan-hMENA Ab (green) and Alexa Fluor conjugated phalloidin (F-Actin, red). Scale bar = 20 µm. Magnification 63X. **(c)** Western blot analysis of BT549 cells transfected with non-targeting siRNA (CNTR) and SRF smart pool siRNAs, with the indicated Abs, showing that SRF silencing reduces β1 integrin expression. **(d)** G-Actin/F-Actin ratio (densitometric data) of BT549 cells transfected with non-targeting siRNA (CNTR) or hMENA(t) siRNA, indicating that hMENA(t) silencing increases the G/F-Actin ratio. Bars represent mean ± SD (n = 3). Representative immunoblot of G and F Actin is reported on the left.

**(e)** Western blot analysis of CNTR and sihMENA(t) BT549 cells with the indicated antibodies. **(f)** Luciferase assay for SRE reporter activity in BT549 cells transfected with non-targeting siRNA (CNTR), sihMENA(t) along with Cignal dual-luciferase SRE Reporter, showing that hMENA(t) silencing significantly reduces the SRF activity. Untransfected BT549 cells treated with the SRF inhibitor, CCG1423 were used as control for the inhibition of SRF activity. Data are reported as the mean ± SD of three independent experiments performed in triplicate. P value was calculated by 2-tailed Student’s t test.

**Supplementary Figure 3.** SRF silencing abrogates the hMENAΔv6 pro-invasive function in cancer cells. Matrigel invasion assay performed on A549 cells (50.000 cells; 24 h of invasion), transfected with non-targeting siRNA (CNTR) or with SRF siRNA and, after 24h, transiently transfected with the empty vector (pcDNA3) or hMENAΔv6. The assay was repeated three times, performed in triplicate each time. Standard deviations are indicated. P value was calculated by 2-tailed Student’s t test. Representative phase-contrast microscopy images of cells are reported. Right: WB analysis of the A549 cells to verify SRF silencing and hMENAΔv6 transfection.

**Supplementary Figure 4.** hMENA(t) silencing reduces β1 integrin activation in hMENA/hMENAΔv6 expressing BT549 and A549 cells. **(a)** Immunofluorescence analysis showing that hMENA/hMENAΔv6 isoforms (green) colocalize with active β1 integrin (9EG7, extended-conformation, red) which is significantly reduced by hMENA(t) silencing in BT549 cells. The quantification of Pan-hMENA/activated β1 integrin colocalization was carried out using the Pearson's correlation coefficient (R). Magnification 100X. Scale bar = 30 µm. **(b)** Quantification of hMENA(t) fluorescence intensity in β1 integrin clusters areas was compared to that in the whole cell to evaluate enrichment of hMENA(t) in the β1 integrin clusters. **(c)** Representative histograms of flow cytometric staining for 9EG7 in BT549 cells transfected with non-targeting (CNTR) or hMENA(t) siRNAs. The percentage of positive cells is shown. **(d)** Percentage of active β1 integrin (9EG7) with respect to total β1 integrin (TS2-16, belt and extended-conformation) analyzed by flow cytometry in BT549 and A549 cells transfected with non-targeting (CNTR) or hMENA(t) siRNAs. Data are reported as the mean ± SEM of three independent experiments. P value was calculated by 2-tailed Student’s t test. **(e)** Percentage of active β1 integrin (9EG7) with respect to total β1 integrin (TS2-16) analyzed by FACS in BT549 cells transfected with non-targeting (CNTR), or VASP siRNAs. Data are reported as the mean ± SD of three independent experiments. **(f)** Western blot analysis of CNTR, sihMENA(t), siVASP BT549 cells, with the indicated Abs.

**Supplementary Figure 5.** hMENAΔv6 transfection increases the expression of P-FAK, P-Paxillin and P-TALIN. (**a)** Representative confocal images of P-FAK (red) of A549 cells transfected with hMENAΔv6-GFP (green). Right:, merge and selected regions of interest (ROI). Average of P-FAK fluorescence intensity in hMENAΔv6-GFP negative or positive cells is shown in the right panel. **(b)** Confocal immunofluorescence images of P-Paxillin (red) of A549 cells transfected with hMENAΔv6-GFP (green). Right: merge and selected ROI. Average of P-FAK fluorescence intensity in hMENAΔv6-GFP negative or positive cells is shown in the right panel. Magnification 63X. Scale Bar = 20 µm. **(c)** WB analysis of DAL CNTR and hMENAΔv6 transfected cells, with the indicated antibodies. **(d)** WB analysis of A549 CNTR and hMENAΔv6 transfected cells, with the indicated antibodies.

**Supplementary Figure 6.** Transfection of the splicing regulator ESRP1 or of hMENA^11a^ reduces the phosphorylation of FAK, SRC, Paxillin and AKT. **(a)** WB analysis of BT549 CNTR cells, hMENA^11a^ transfected cell clone (#104) or ESRP1 (which includes the 11a exon) transduced cells, with the indicated antibodies. **(b)** Confocal analysis with the indicated antibodies of BT549 CNTR cells and hMENA^11a^ expressing clone #104 with Pan-hMENA (green) and P-Paxillin (red) Abs. Magnificatio 63X. Scale bar = 20 µm.

**Supplementary Figure 7.** **(a)** Representative Western blots of the conditioned medium (upper panel) or of the whole cell lysates (lower panel) of DAL cells transfected with empty vector (CNTR), hMENA^11a^ and hMENAΔv6, with the indicated Abs. **(b-c)** Fold increase or reduction of FN1 protein expression in hMENAΔv6 or hMENA^11a^ transfected cells with respect to control cells. Densitometric quantification was determined by using ImageJ. Data are reported as the mean ± SD of three independent experiments. P value was calculated by 2-tailed Student’s t test. **(d)** qRT-PCR analysis of FN1 mRNA expression in the DAL cells transfected with control (CNTR), hMENA^11a^ and hMENAΔv6. Data are reported as the mean ± SD of three independent experiments. P value was calculated by 2-tailed Student’s t test. **(e)** Percentage of active β1 integrin (9EG7) with respect to total β1 integrin (TS2-16) analyzed by flow cytometry in BT549 CNTR (hMENA^11a^ negative/hMENAΔv6 positive) or hMENA^11a^ expressing clones, cultured on uncoated or for 1h on FN1 coated wells. Data are reported as the mean ± SD of three independent experiments. P value was calculated by 2-tailed Student’s t test. Ns: not significative.
