# Supplementary material for: hMENA isoforms impact NSCLC patient outcome through fibronectin/β1 integrin axis
Source: Oncogene. 2018 Jun 15;37(42):5605–17. doi: 10.1038/s41388-018-0364-3 (PMC6193944; doi:10.1038/s41388-018-0364-3)
Supplement: Supplementary file 5 — Supplementary Table 2 [file 41388_2018_364_MOESM5_ESM.doc]

**Supplementary Table 2. Clinico-pathological characteristics of the NSCLC cancer patients**

| **CHARACTERISTICS** | **No of cases** | **%** |
| --- | --- | --- |
| **Number of patients** | **114** |  |
| **Age at diagnosis** |  |  |
| Median (range) | 68 yrs (28-83) |  |
| **Sex** |  |  |
| Male | 83 | 73 |
| Female | 31 | 27 |
| **Histotype** |  |  |
| Adenocarcinoma | 62 | 54 |
| Squamous carcinoma | 41 | 36 |
| Other | 11 | 10 |
| **Lymph node** |  |  |
| N- | 114 | 100 |
| N+ | 0 | 0 |
| **Grading** |  |  |
| Grade 1 | 2 | 2 |
| Grade 2 | 51 | 45 |
| Grade 3 | 60 | 52 |
| Unknown | 1 | 1 |
| **Tumor size** |  |  |
| T1 | 37 | 33 |
| T2 | 58 | 51 |
| T3 | 14 | 12 |
| T4 | 4 | 3 |
| Unknown | 1 | 1 |
| **HybMENA^11a^** |  |  |
| Negative | 66 | 58 |
| Positive (hMENA^11a^ High/hMENA(t) Low) | 48 | 42 |
| **Stromal Fibronectin** |  |  |
| Low (0-1) | 51 | 45 |
| Positive (2-3) | 63 | 55 |
